# Development of a feline model for preclinical research of a new translabyrinthine auditory nerve implant

**DOI:** 10.3389/fnins.2024.1308663

**Published:** 2024-02-06

**Authors:** W. Mitchel Thomas, Steven A. Zuniga, Inderbir Sondh, Moritz Leber, Florian Solzbacher, Thomas Lenarz, Hubert H. Lim, David J. Warren, Loren Rieth, Meredith E. Adams

**Affiliations:** ^1^Department of Biomedical Engineering, University of Utah, Salt Lake City, UT, United States; ^2^Department of Otolaryngology-Head and Neck Surgery, University of Minnesota, Minneapolis, MN, United States; ^3^Department of Biomedical Engineering, University of Minnesota, Minneapolis, MN, United States; ^4^Blackrock Neurotech, Salt Lake City, UT, United States; ^5^Department of Electrical and Computer Engineering, University of Utah, Salt Lake City, UT, United States; ^6^Department of Otorhinolaryngology, Medical University of Hannover, Hannover, Germany; ^7^Department Mechanical and Aerospace Engineering, West Virginia University, Morgantown, WV, United States

**Keywords:** auditory nerve implant, Utah electrode array, translabyrinthine approach, feline, cat, preclinical model, auditory prostheses, nerve stimulation

## Abstract

Cochlear implants are among the most successful neural prosthetic devices to date but exhibit poor frequency selectivity and the inability to consistently activate apical (low frequency) spiral ganglion neurons. These issues can limit hearing performance in many cochlear implant patients, especially for understanding speech in noisy environments and in perceiving or appreciating more complex inputs such as music and multiple talkers. For cochlear implants, electrical current must pass through the bony wall of the cochlea, leading to widespread activation of auditory nerve fibers. Cochlear implants also cannot be implanted in some individuals with an obstruction or severe malformations of the cochlea. Alternatively, intraneural stimulation delivered via an auditory nerve implant could provide direct contact with neural fibers and thus reduce unwanted current spread. More confined current during stimulation can increase selectivity of frequency fiber activation. Furthermore, devices such as the Utah Slanted Electrode Array can provide access to the full cross section of the auditory nerve, including low frequency fibers that are difficult to reach using a cochlear implant. However, further scientific and preclinical research of these Utah Slanted Electrode Array devices is limited by the lack of a chronic large animal model for the auditory nerve implant, especially one that leverages an appropriate surgical approach relevant for human translation. This paper presents a newly developed transbullar translabyrinthine surgical approach for implanting the auditory nerve implant into the cat auditory nerve. In our first of a series of studies, we demonstrate a surgical approach in non-recovery experiments that enables implantation of the auditory nerve implant into the auditory nerve, without damaging the device and enabling effective activation of the auditory nerve fibers, as measured by electrode impedances and electrically evoked auditory brainstem responses. These positive results motivate performing future chronic cat studies to assess the long-term stability and function of these auditory nerve implant devices, as well as development of novel stimulation strategies that can be translated to human patients.

## 1 Introduction

Cochlear implants (CIs) are effective neural prosthesis for restoring hearing to those with severe to profound sensorineural hearing loss (Zeng, 2017; Naples and Ruckenstein, 2020). However, the performance of CIs is poor in noisy environments and with competing sound sources such as multiple speakers (Stickney et al., 2004; Tobey et al., 2011). The ability to appreciate music is also limited among CI users (McDermott, 2004). These limitations stem from the poor selectivity of stimulated auditory fibers and the inability to recruit low-frequency fibers from the apex of the spiral ganglion. A significant source of these deficiencies is the poor electrode-tissue interface for cochlear electrodes. A poorly conductive material (i.e., bone) separates the electrodes from the spiral ganglion, and the highly conductive perilymph within the cochlea results in shunting paths (Zeng et al., 2014; Zeng, 2017). These environmental factors around the CI cause undesirable current spread, which leads to the activation of a relatively large extent of the spiral ganglion. This limits the ability of the CIs to selectively activate specific frequency regions of the cochlea, despite following the tonotopic structure of the cochlea. As CI electrodes typically do not reach beyond the middle turn of the cochlea, the implant cannot consistently activate low-frequency fibers located in the apex of the cochlea. These fibers play a major role in speech perception (Skinner et al., 2002; Wardrop et al., 2005).

A promising alternative to CI stimulation is direct stimulation of the auditory nerve using penetrating electrodes. Direct stimulation of the auditory nerve was performed chronically in humans using stainless-steel wires in the mid-1960s in the United States (Simmons, 1966). Simmons' studies demonstrated that auditory percepts could be evoked from microwire stimulation in human subjects (Simmons, 1983), opening the door for further exploration into auditory nerve implant (ANI) stimulation. In Germany in the late-1970's, the Naumann et al. (1986) and Zwicker et al. (1986) also chronically implanted electrode wires into the human auditory nerve which showed encouraging perceptual results over the course of a two-month implant period.

There were multiple groups who investigated the development and functional effects of an ANI in animal models from the 1980's onwards using a range of electrode materials (Arts et al., 2003). One of the technologies and approaches relevant for our study occurred in the mid-2000s, when the Normann group at the University of Utah implanted Utah Electrode Arrays (UEAs) (Blackrock Neurotech, Salt Lake City, UT, USA) into the auditory nerve exposed through the modiolus of the feline cochlea (Badi et al., 2002). Badi et al. (2003) and Hillman et al. (2003) found that intraneural stimulation through individual microelectrodes of the UEA could evoke auditory brainstem responses and reported microelectrode stimulation thresholds from 5 to 100 μA, which are much lower than possible with CIs. They also demonstrated the ability to activate different populations of neurons (Badi et al., 2007; Kim et al., 2007). Later in the late 2000s, Middlebrooks and colleagues investigated intraneural stimulation of the cat auditory nerve with a single-shank NeuroNexus (Ann Arbor, MI, USA) electrode array in the modiolus coupled with recordings from the inferior colliculus. Middlebrooks and Snyder (2007, 2008) found that direct stimulation of the auditory nerve conferred a higher degree of frequency-specific activation and at lower stimulation levels when compared to CIs. This group also demonstrated that electrode implantation within the nerve enables direct access to and robust activation of fibers that encode low-frequency sounds (Middlebrooks and Snyder, 2007).

These various ANI approaches can better leverage the tonotopy of the auditory nerve than CIs via the modiolus or near the spiral ganglion (Sando, 1965). Furthermore, ANIs offer an alternative hearing restoration solution for patients who are poor candidates for a CI due to abnormal cochlear anatomy, ossification of the cochlea, undesired facial nerve activation, or other contraindications. While alternative auditory prostheses are being explored, such as auditory midbrain and auditory brainstem implants, these implants are designed more for those who do not have a functional auditory nerve and require brain surgery for a hearing prosthesis (Lim and Lenarz, 2015; Wong et al., 2019). The wealth of studies demonstrating the potential of ANIs supports further research to identify new hearing prosthesis approaches targeting the auditory nerve.

A key innovation since the prior ANI studies is the refinement of new penetrating electrode array designs for stimulating and recording of peripheral nerves. In particular, the studies by the Normann group described above utilized 4×4 UEAs, whose uniform-length microelectrodes are generally geared toward cortical studies. Their UEA design likely only gave access to a portion of the cross-sectional area of the auditory nerve and prevented recruitment of the entire tonotopy of the nerve. In recent years, specialized slanted versions of the UEA designed specifically for peripheral nerve implantation have been developed (Branner et al., 2001). Rather than a uniform shaft length, these Utah Slanted Electrode Arrays (USEAs) consist of a grid of penetrating microelectrodes with different shaft lengths along the columns, giving the device access to the majority of the cross-section of a nerve. The USEA device has demonstrated a high degree of selectivity for the activation of small populations of axons in peripheral nerves. Stimulation through USEAs has allowed for graded recruitment of independent motor units and selective activation of distinct sensory precepts in pre-clinical animal experiments and clinical human studies (Branner et al., 2004; McDonnall et al., 2004; Normann et al., 2012; Ledbetter et al., 2013; Davis et al., 2016; Wendelken et al., 2017; George et al., 2020). We hypothesize that the use of slanted UEA variants in the auditory nerve will lead to higher selectivity compared to prior ANI studies.

Building upon the successes of prior ANI studies and approaches, this report describes the development of a new transbullar translabyrinthine surgical approach (referred to as translabyrinthine hereafter) geared toward the successful implantation of a novel ANI device, leveraging the USEA technology. Since the USEA technology has not yet been investigated for an ANI application, initial experiments were required to develop an appropriate surgical approach and proper implantation techniques (e.g., nerve insertion and cable anchoring methods) to demonstrate the feasibility of a feline animal model for this new technology. This report presents photographic images of cadaveric and acute studies used in our development of the translabyrinthine approach for the USEA-based ANI. It also presents electrode impedance data and evoked auditory brainstem response (eABR) recordings in response to ANI stimulation to demonstrate the success of our implantation approach. Overall, the results support that the cat animal model has potential for use in preclinical studies for ANI development and that implantation of the USEA auditory nerve using our translabyrinthine approach can lead to positive functional results, paving the way for future chronic ANI studies to confirm safety and selectivity of activation across the tonotopic organization of the auditory pathway for hearing restoration.

## 2 Methods

### 2.1 Animal model and skull base approach selection

**Figure 1:**
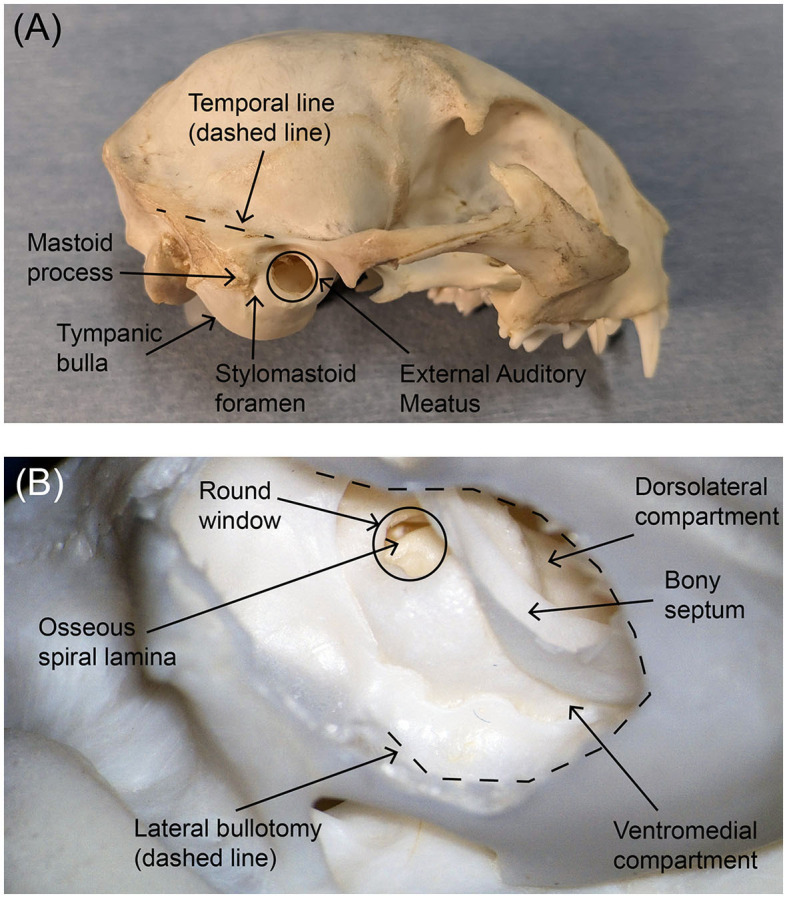
Photographs of **(A)** the external lateral view of the feline skull and **(B)** the interior posteroinferior view of the tympanic bulla after bullotomy. Key anatomical markers are labeled. The area of the tympanic bulla that will be removed during an ANI procedure is traced with a black dotted line in the lower photograph. In both panels, rostral is to the right.

Feline models are the dominant large animal model for hearing prosthetics research and have been used extensively for CI research (an example of the cat skull with key anatomical markers labeled is shown in Figure 1). These CI studies, as well as other scientific studies, have produced a robust volume of literature detailing the anatomy and physiology of the cat auditory nerve and temporal bone as well as the electrophysiologic characteristics of their auditory pathways (Silverstein, 1972; Clark et al., 1975; Davey, 1979; Hartmann et al., 1984; Little and Lane, 1986; Leake and Snyder, 1989; Hatsushika et al., 1990). Thus, the cat model represents one potential preclinical model for developing an ANI surgical approach and determining the electrophysiological and auditory psychophysical characteristics of the ANI. In particular, the existence of frequency-specific mapping of the cat auditory nerve (Sando, 1965; Arnesen and Osen, 1978) suggests this model can help evaluate the performance of our devices as well as their safety and efficacy. Functionally, the cat auditory nerve has similar diameter to the human auditory nerve, allowing us to use and evaluate the same USEA design in these studies as will be used with human subjects (Badi et al., 2002, 2003).

The importance of cats for pre-clinical large animal studies involving auditory prostheses and psychophysics and the strong interest in intraneural approaches to advancing hearing prosthetics provide strong motivation for our study. The optimal surgical approach for implantation of the cat auditory nerve was developed in two stages. First, systematic microdissections of cat cadavers were performed to evaluate several individual and combined approaches. These approaches included transbullar, retrolabyrinthine, translabyrinthine, retrosigmoid, and transcochlear approaches, each based on modifications of established human surgical skull base approaches to the internal auditory canal (IAC). Second, a series of acute pilot experiments (i.e., non-recovery surgeries with anesthetized cats) to expose the auditory nerve and implant the ANI were performed. These studies were performed to evaluate the feasibility of the surgical approaches, refine the preferred approaches, and evaluate the efficacy of arrays implanted by the chosen procedure.

### 2.2 Anatomical evaluation

Our initial work involved evaluating the current scholarly knowledge pertaining to neurophysiology of the feline auditory nerve and visualization of the associated anatomy and neuroanatomy. This work focused on (1) developing a model that best represented the leading human surgical approaches, particularly the translabyrinthine and retrosigmoid approaches, and (2) evaluating alternative approaches to generate a robust surgical approach to the auditory nerve. The team carefully reviewed the literature, particularly for prior studies using microelectrode arrays (Badi et al., 2002, 2003; Hillman et al., 2003; Middlebrooks and Snyder, 2007, 2008). We also utilized anatomical handbooks (Gilbert, 1975), and online resources with labeled CT imagery of the appropriate skull base regions (Silverstein, 1972; Davey, 1979; Little and Lane, 1986).

### 2.3 Cadaveric surgical studies

Seven cat cadaver experiments were performed to evaluate existing and new approaches to access the cat auditory nerve. The feasibility of neural implantation of the prosthesis was determined by examining if the approach could potentially avoid stimulation of the adjacent vestibular and facial nerves.

### 2.4 Electrode array specifications

**Figure 2:**
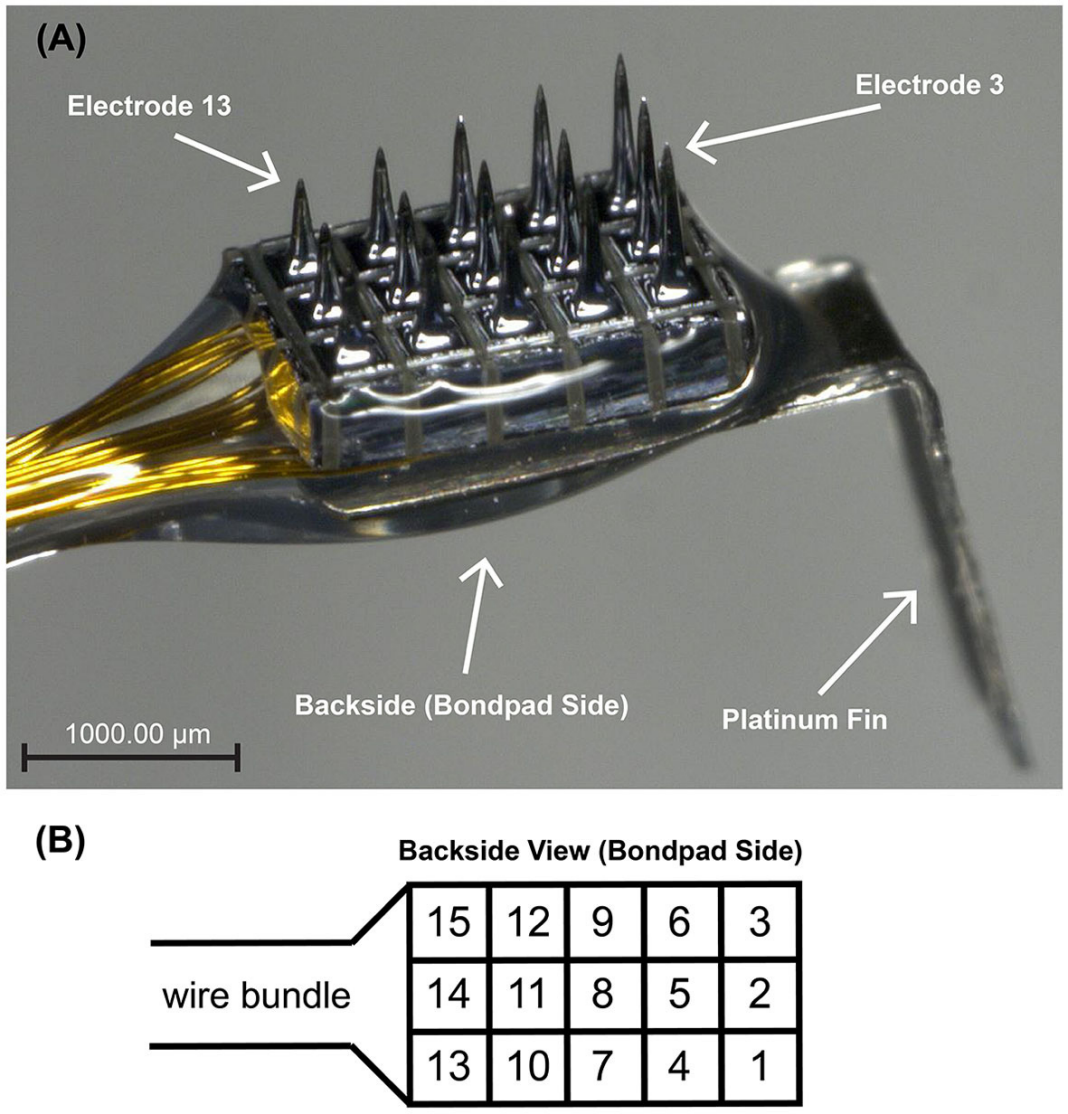
**(A)** Micrograph of the 3×5 ANI USEA of the second design. Going along a column, shaft lengths progress from 0.5 to 0.9 mm. The platinum fin at the end of the device facilitates surgical handling during implantation procedures. A 1,000 μm scale bar is shown at the bottom left. Electrodes 3 and 13 and the back side of the array are indicated for orientation purposes. **(B)** is a schematic representation of the ANI USEA from the back side of the array (i.e., bondpad side) with numbered electrodes.

Based upon measurements of the exposed auditory nerve and the distance to the frontal bone overlying the frontal sinus in the first seven cadaver procedures, customized USEAs were developed. Custom USEAs with two different designs were manufactured by Blackrock Neurotech (Salt Lake, UT). A micrograph of the second design is shown in Figure 2. Both array designs had a 3×5 shaft layout, with each row having three electrodes of the same length. Each of the five columns contained shanks of progressively longer lengths. Shaft length progressions included either 0.4–1.3 mm (first design, used with the first nine acute cats) or 0.5–0.9 mm (second design, used with the last four acute cats). Both designs could be fully implanted into the feline auditory nerve, which based on our own measurements and prior studies, has an approximate diameter of 1.5 mm (Badi et al., 2002). Electrode shanks had the same 400 μm pitch as previous UEAs and USEAs. The pointed end of each shank is the electrode, which is metalized with sputtered iridium oxide films (SIROF) with an allowed tip exposure tolerance between 50 μm and 100 μm regardless of shaft length. The remainder of each shank, and the rest of the USEA, is insulated with Parylene-C with overlap between the SIROF and Parylene-C layers to ensure that no silicon is exposed. The second design also added a 2-mm vertical platinum fin attached to the bondpad side of the array to improve handling of the device during surgery and placement on the nerve. Each array was attached to a Cereport connector by an 85 mm wire bundle containing the 15 insulated gold alloy wires corresponding to each electrode, along with 2 Pt-Ir wires to increase the stiffness and formability of the lead. The first 80 mm of the wire bundle starting from the connector was arranged using helically wound and silicone overmolded wires with a 1.3 mm diameter. The distal 5 mm of the bundle used linearly arranged wires and was hand potted to facilitate bending of the lead for angled insertion of the device and to maintain gentle pressure holding it in the nerve.

### 2.5 Non-survival cat studies

A total of thirteen felines were utilized in non-survival studies, with eight procedures at the University of Utah and five procedures at the University of Minnesota. These studies were used to develop the surgical approach and optimize the device configuration. The studies were conducted with approval from the Institutional Animal Care and Use Committees (IACUC) from the respective institutions. Nine procedures focused on the development of the translabyrinthine approach to access the IAC in a live animal preparation. Impedance measurements and electrophysiological measurements and analyses were performed as part of the remaining four procedures. *In vivo* impedance measurements were used to evaluate damage to the USEA during surgical handling and to quantify the increase in impedance in the implantation environment compared with saline. The eABR measurements were used to confirm engagement with the auditory nerve and its functionality after implantation.

All felines were fasted 12–18 h before the procedure and received pre-operative dexamethasone sodium phosphate (0.5 mg/kg IM) to limit edema. At least 2 h before surgery, buprenorphine (0.01–0.03 mg/kg) was administered as an analgesic. Anesthesia was induced using Telazol (9–13 mg/kg) and maintained using isoflurane (0.5%–5%) in O_2_ after intubation. IV lines were placed and sterile lactated Ringer's solution was delivered continuously throughout the surgical procedure (8–12 ml/kg/h). Felines were intubated and received atropine (0.02–0.74 mg/kg IV) to control tracheal secretions. An additional dose of dexamethasone sodium phosphate (0.5 mg/kg IV) was administered peri-operatively. Felines were administered either cefazolin (20–25 mg/kg IV) or ampicillin/sulbactam (22–30 mg/kg IV) systematic antibiotics every 90–120 min following the initial incision. Felines were either mechanically ventilated for the entire procedure or allowed to breathe independently under sufficient anesthetic depth. Rectal temperature, tidal volume, end-tidal CO_2_, SpO_2_, and respiration rate were monitored and recorded every 15 min, and body temperature was maintained through a heated water blanket. The surgical procedures were limited to 10 h, which included exposure of the auditory nerve and implantation of the ANI USEA with the long electrodes proximal to the modiolus (details provided in the next section). The electrode array was characterized and electrophysiological recordings performed in the last four animals. Upon conclusion of the acute study, the felines were euthanized with either sodium pentobarbital (85 mg/kg IV) or potassium chloride (2 mEq/kg or 150 mg/kg IV) while under isoflurane anesthesia.

### 2.6 Impedance testing and eABR acquisition

Impedance measurements from all ANI USEA electrodes were taken in three of the four non-survival animal implants included in this work. Impedance data were not collected on the fourth implant due to exhaustion of the time allotted by our protocol (10 h). These measurements were used to evaluate the condition of the electrode arrays in saline prior to implantation and after implantation in the physiological environment. Impedance magnitudes were recorded using a custom-built Automated Impedance Testing (AIT) system designed for Utah electrode arrays (Gunalan et al., 2009). The AIT system uses a custom-made potentiostat to drive the test solution or animal with a 0.2 s, 1 kHz sinewave of 10 mV amplitude (zero to peak) and measures the current flowing through one electrode of the USEA that is attached to the stimulus return. The potentiostat counter and reference electrodes are Pt wires placed in the solution or tissue of the animal. One electrode of the ANI USEA serves as the working electrode. Electrodes are tested one at a time with relays used to switch between the ANI USEA electrode being tested. The impedance magnitude is calculated by the LabVIEW Frequency Response Function vi using the voltage as inputs at the reference electrode and the current flowing through the working electrode. Both the reference electrode voltage and the working electrode current were sampled at 128 kHz. The impedances reported herein are described as the unshunted impedance (or tip impedance) in Gunalan et al. (2009). This impedance is an estimate of the true impedance of the electrode after the removal of all inter-electrode shunting impedance pathways. Saline soak testing was performed for one week with the impedance of each electrode measured at least once per day. Impedance values from the final five days of soak testing were used for statistical analysis. The impedance of implanted electrodes was also measured with the AIT, with five sequential impedance measurements performed for each electrode following implantation.

Statistical analysis was performed on the mean *in-vitro* (saline) and *in-vivo* (implant) impedances. Prior to statistical analysis, any electrode that had an impedance value above 500 kΩ in either *in-vitro* or *in-vivo* testing was removed. The *in-vitro* and *in-vivo* values for electrodes on each array were treated as paired samples, tested for normality using the Shapiro–Wilk test, and the difference was evaluated using a paired t-test. An additional statistical analysis (one-way ANOVA) was performed to determine if there was a difference in impedance between electrodes of different shaft lengths. Both statstical tests were performed using GraphPad Prism version 10.0.0 (GraphPad Software, Boston, Massachusetts USA). Log_10_ scaling was used for plotting to provide better visualization as a violin plot.

eABRs were collected as a function of current stimulation using the implanted USEAs. These experiments evaluated the functional effects of auditory nerve stimulation and the ability of the USEA to engage the auditory nerve fibers. They can also identify off-target effects (e.g., facial nerve or vestibular nerve stimulation). Due to procedural time constraints, only a subset of likely candidate channels was examined in each implant, though recordings at multiple stimulus intensities were possible in the final feline experiment. Stimulation was delivered using either an IZ2 stimulator [Tucker Davis Technologies (TDT), Alachua, FL] or a Subject Interface Module (SIM) including IZ10 stimulators (TDT, Alachua, FL). Alternating cathodic-first and anodic-first biphasic stimulation pulses were delivered at a stimulation frequency of 8–10 Hz. Pulse durations were 100 μs per phase, and intensities varied between 0 and 150 μA. Commercial subdermal needle electrodes (LifeSync Neuro, Coral Springs, FL) were used to collect eABR recordings and consisted of recording, reference, and ground contacts. One recording electrode was placed in each of the ipsilateral and contralateral mastoid regions (contralateral data was used for secondary validation and the data are not provided). The reference electrode was placed on the vertex of the head and the ground electrode was placed in the nape. Differential recordings between the recording and reference electrodes were obtained using either a RA4LI low impedance head stage (TDT, Alachua, FL) connected to a LabRat front-end (TDT, Alachua, FL) or a Medusa4Z bioamp connected to an RZ6 (TDT, Alachua, FL). The RA4LI had a hardware highpass filter corner of 2.2 Hz and a lowpass filter corner of 7.5 kHz with an input voltage range of ±80 mV. The Medusa4Z had a hardware highpass filter of 0.3 Hz and a lowpass corner 11.25 kHz with an input voltage range of ±10 mV. All neural recordings were sampled at 25 kHz.

## 3 Results

### 3.1 Cadaveric findings

**Figure 3:**
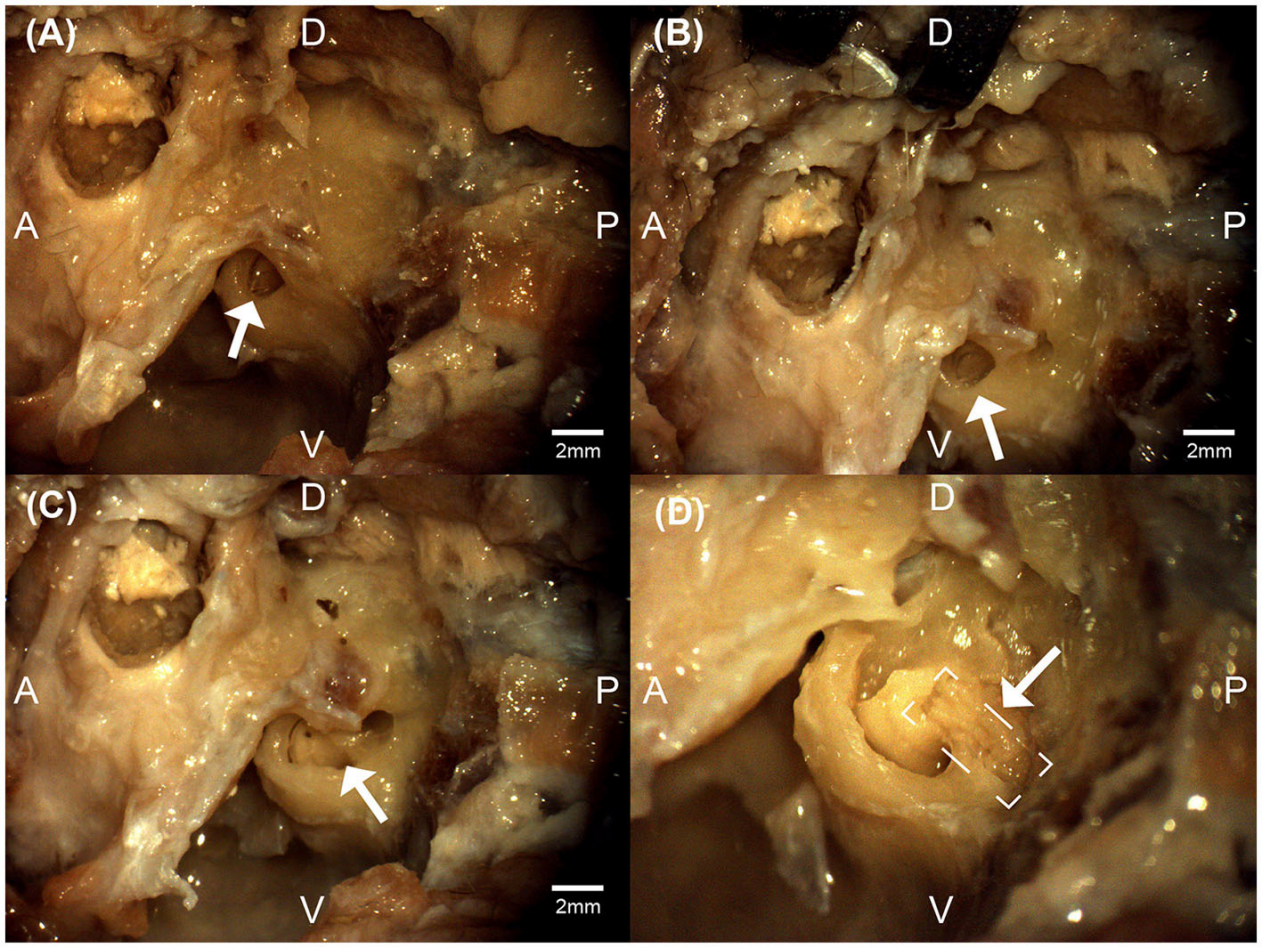
Micrographs from the left bulla region of a feline cadaver exploring the transbullar translabyrinthine approach. The white arrows denote the exposed round window niche **(A)**, the exposed cochlea **(B)**, the exposure of the modular region **(C)**, and the exposure of the auditory nerve (boxed) within the modiolar region **(D)**. Anatomical directions for posterior [P], anterior [A], dorsal [D], and ventral [V] are indicated. Scale bars for **(A–C)** are estimated using feline cadaveric skulls' round window niche diameter. **(D)** is at higher magnification.

The advantages and disadvantages of different approaches to the auditory nerve in a cat model were considered. The intracranial retrosigmoid approach that can be used for auditory brainstem implants permits vestibular labyrinth preservation. This requires significant cerebellar compression and aspiration, resulting in an unacceptable degree of morbidity. The retrosigmoid approach also offered suboptimal visualization and access to the lateral auditory nerve between the internal meatus and cochlear nucleus (McCreery et al., 1992, 2000, 2013). Similarly, the transbullar retrolabyrinthine approach preserves the labyrinth but also afforded limited exposure of the auditory nerve. While the transmodiolar approach allowed for enough auditory nerve exposure to facilitate implantation, associated trauma to the modiolus, and thus Rosenthal's canal, resulted in an unacceptable degree of injury to the spiral ganglia for future chronic study. By contrast, the intracanalicular and cisternal segments of the auditory nerve were safely accessed via the transbullar translabyrinthine approach. While the bony vestibular labyrinth is removed to access the internal auditory canal, the translabyrinthine exposure provided adequate visualization and access to the facial nerve and all aspects and segments of the auditory nerve, while preserving the anatomical and physiological integrity of the nerve and spiral ganglion neurons. This access facilitated the implantation of a penetrating nerve array without appreciable contact with the vestibular or facial nerves. An example of auditory nerve exposure in a cadaver at various stages is shown in Figure 3.

### 3.2 Surgical access and findings

The animal was positioned in the lateral position with the head extended and rotated contralaterally by ~30 degrees. A curvilinear incision was fashioned around the base of the pinna, directly lateral to the tympanic bulla. The skin, subcutaneous tissue, platysma, and postauricular muscles were incised. The cartilaginous portion of the external auditory canal is identified and followed medially to the bony rim of the external auditory meatus. The tympanic bulla lies just inferior to the external auditory meatus and was exposed by blunt dissection of the sternomastoid and cleidomastoid muscles. The facial nerve was identified emanating from the stylomastoid foramen and followed anteriorly as it coursed over the lateral surface of the tympanic bulla toward the parotid gland. The facial nerve was meticulously mobilized superiorly to avoid trauma during lateral bullotomy.

A high-speed dental drill was used to perform a lateral bullotomy exposing the smaller dorsolateral compartment of the tympanic bulla. The bony septum separating the larger ventromedial and smaller dorsolateral compartments of the tympanic bulla was removed, allowing for identification of the stapes, oval window, and round window. The bone at the posterior aspect of the tympanic bulla was exenterated using the dental drill with various round cutting and diamond burrs. Continuous suction-irrigation was used to cool the bone and keep the field free of debris. Drilling proceeded medially following the temporal line and posterior aspect of the tympanic bulla to identify the middle fossa dura and lateral venous sinus, respectively. The lateral semicircular canal and posterior semicircular canal are encountered during this dissection and followed medially to the vestibule. The vestibule was opened and the superior and inferior vestibular nerves were identified and followed medially allowing for approximation of the location of the internal auditory canal. The round window was then drilled allowing for exposure of the basal turn of the cochlea. Care was taken to identify and preserve the modiolus. The intervening bone between the modiolus and vestibule was meticulously dissected to expose a segment of the auditory nerve adequate for implantation of the USEA Several bony ledges and bone grooves were created during dissection to allow for anchoring of the wire bundle.

A coronal incision was then made and dissection was carried down to the frontal portion of the calvarium. Using a dental drill with a straight bur, six pilot holes were drilled to accommodate 2-mm diameter, 6-mm length titanium hex anchoring screws (Movora, 310 Commerce Lake Drive, Ste. 107 St. Augustine, FL 32095) for the percutaneous Cereport connector. A subperiosteal tunnel was created to connect the connector site with the lateral bullotomy to accommodate the transmission of the electrode array. A plastic sheath was passed through this tunnel to facilitate transmission of the electrode array from its origin at the connector site to its insertion site at the auditory nerve.

ANI USEA insertion was accomplished utilizing either a custom pneumatic insertion device or manual insertion. The custom pneumatic inserter is similar to the standard Blackrock Neuroport Array Inserter, the development of which was previously presented but with a longer insertion shaft to facilitate access within the surgical field (Rousche and Normann, 1992; Thomas et al., 2022). Confirmation of full insertion of all tines of the USEA was confirmed by observation with the surgical microscope. A fascial graft was placed around the implant after insertion. Incisions were closed in layers. For a subset of studies, the cat was then prepared the cat was then prepared for auditory electrophysiologic studies.

**Figure 4:**
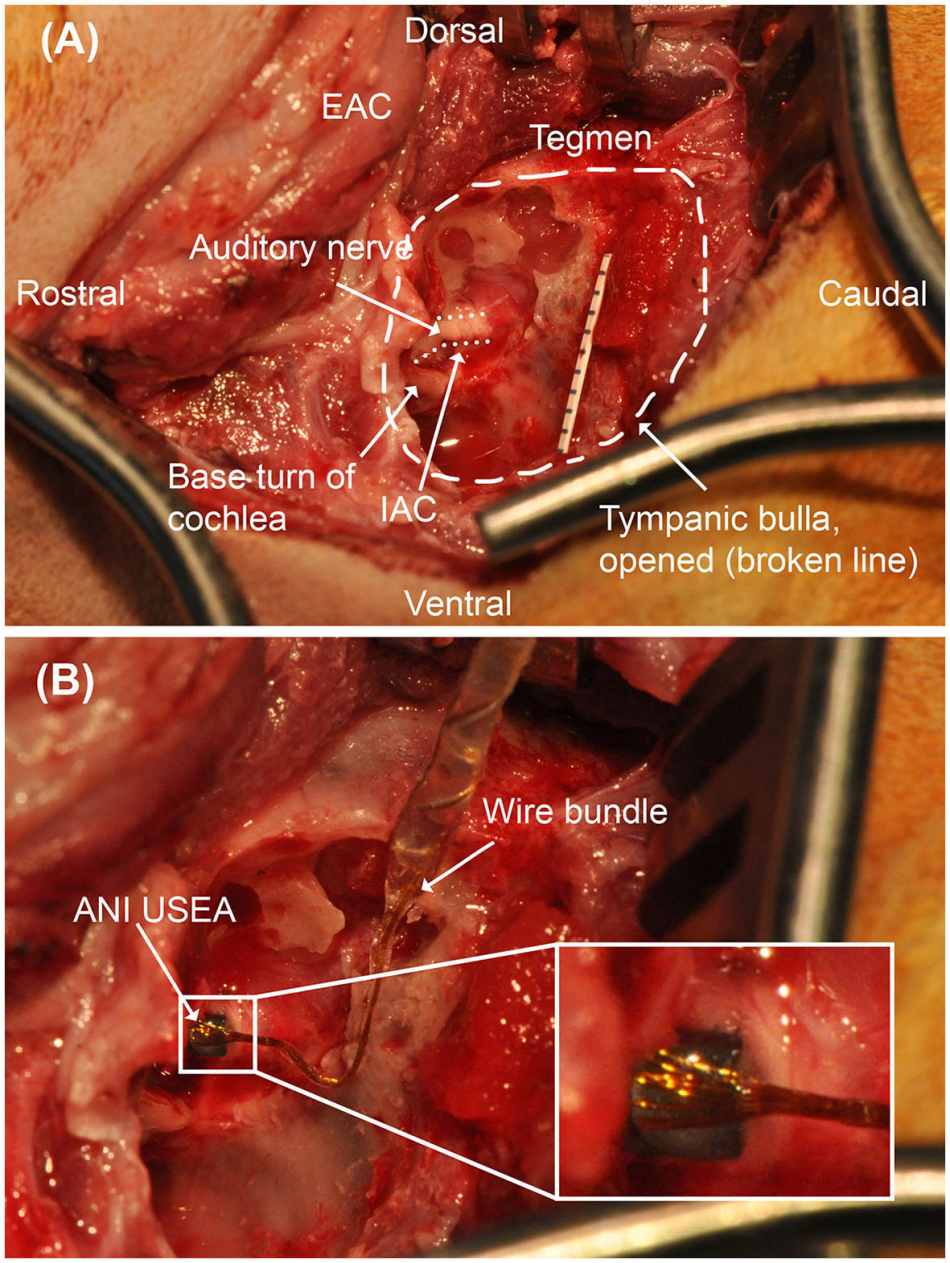
Operative microscopy views of the translabyrinthine ANI procedure. **(A)** Surgical landmarks used to identify the auditory nerve, including the tegmen and basal turn of the cochlea are identified. Millimeter paper is placed in the field for scale. The auditory nerve target is depicted with a dotted line border. **(B)** 3 × 5 ANI USEA implanted into the auditory nerve. IAC, internal auditory canal. The inset box shows a close-up view of the implanted ANI USEA.

The translabyrinthine approach allowed for ready identification of important surgical landmarks used in the identification of the internal auditory canal and auditory nerve. Damage to surrounding neural structures, including the facial nerve, was not visually observed in any procedure. Figure 4A demonstrates an operative microscopy view of the translabyrinthine procedure, demonstrating key anatomic landmarks including the tegmen, internal auditory canal, and basal turn of the cochlea. A successful implant of an ANI USEA is shown in Figure 4B. No hemorrhaging from the auditory nerve was observed after the implant at up to 300× magnification in any of the procedures. The clarity of these landmarks allows for this procedure to be performed by skull base surgeons familiar with feline skull base anatomy.

Similar to cochlear implantation, bony ledges and grooves were created within and around the tympanic bulla to facilitate anchoring of the electrode array. This included a 1 mm notch in the area of the temporal line as well as residual bone from the lateral wall of the tympanic bulla. A fascial sheath was also fashioned and positioned around the inserted electrode array to reinforce the direct apposition between the electrode array and auditory nerve.

### 3.3 Electrophysiological findings

**Figure 5:**
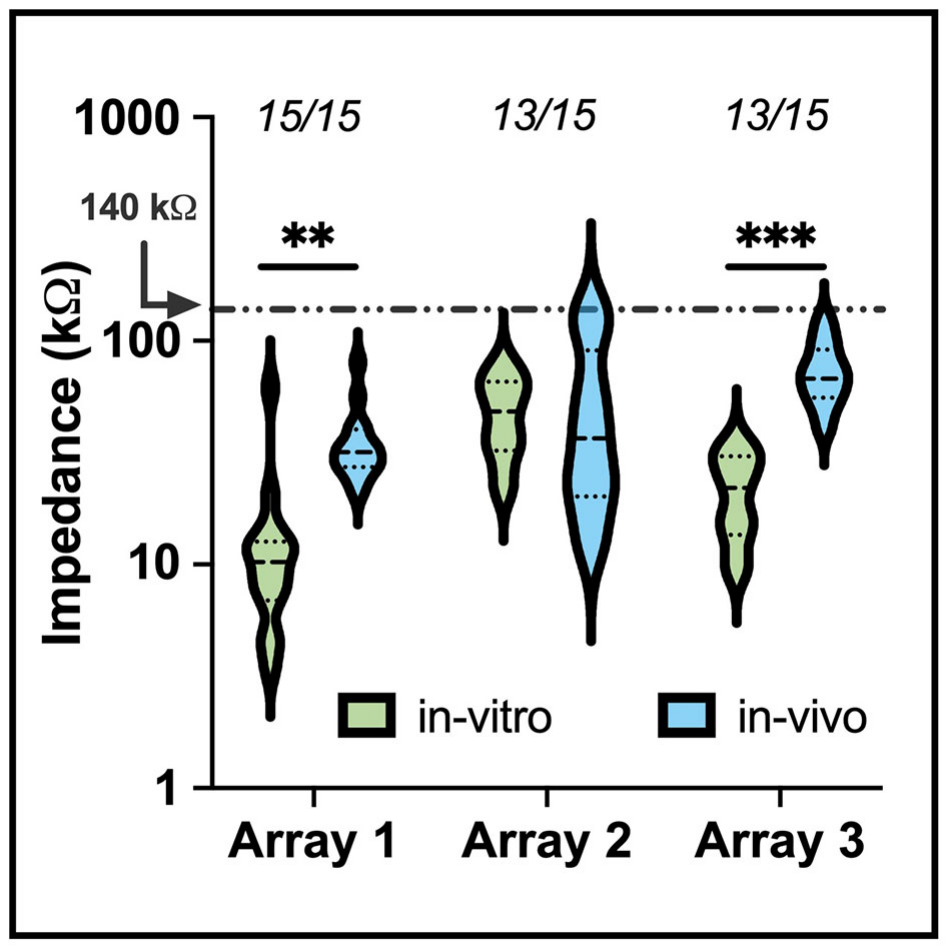
A violin plot comparing *in-vitro* (saline) and *in-vivo* (implant) electrode impedances of three of the devices used for electrophysiology in the study. The median (black dashed) and interquartile range (black dotted) of electrode impedance increased following implant but remained within expected values for electrodes within the tissue. The numbers above each set indicate the number of electrodes below 500 kΩ. Electrodes that presented an impedance value above 500 kΩ were removed from the plot. The shift in *in-vitro* and *in-vivo* impedance was statistically significant for arrays 1 and 3 (^**^p < 0.01, ^***^p < 0.001).

Figure 5 shows the impedance data collected for each electrode array during *in-vitro* soak tests and *in-vivo* measurements after implantation into the auditory nerve. Impedance values recorded during the soak tests varied, but did not indicate broken or nonfunctional electrodes as defined by other studies (George et al., 2020). The variability in soak impedance is within the normal range observed in manufacturing. A mean increase of 26.34 ± 4.78 kΩ (mean ± standard error) in impedance was observed following implantation, which is comparable to impedance shifts between saline and tissue recordings seen in prior studies for other clinical applications (Kane et al., 2013; Black et al., 2018; Gardner et al., 2018). There was no significant difference between the impedance's of electrodes grouped by shaft length (one-way ANOVA, *p* = 0.98). High (>500 kΩ) impedance values, which are indicative of electrode damage, shaft breakage, or wire breakage, were observed on two electrodes on the second and third array, suggesting that >80% of electrodes survived implantation. All unbroken electrode sites had impedances below 140 kΩ, enabling these sites the potential to provide adequate stimulation current (50 μA) to evoke neuronal activity, i.e. being within the compliance limit of the clinical cochlear simulators developed by MEDEL, which is our partner for developing a future human-grade ANI device.

**Figure 6:**
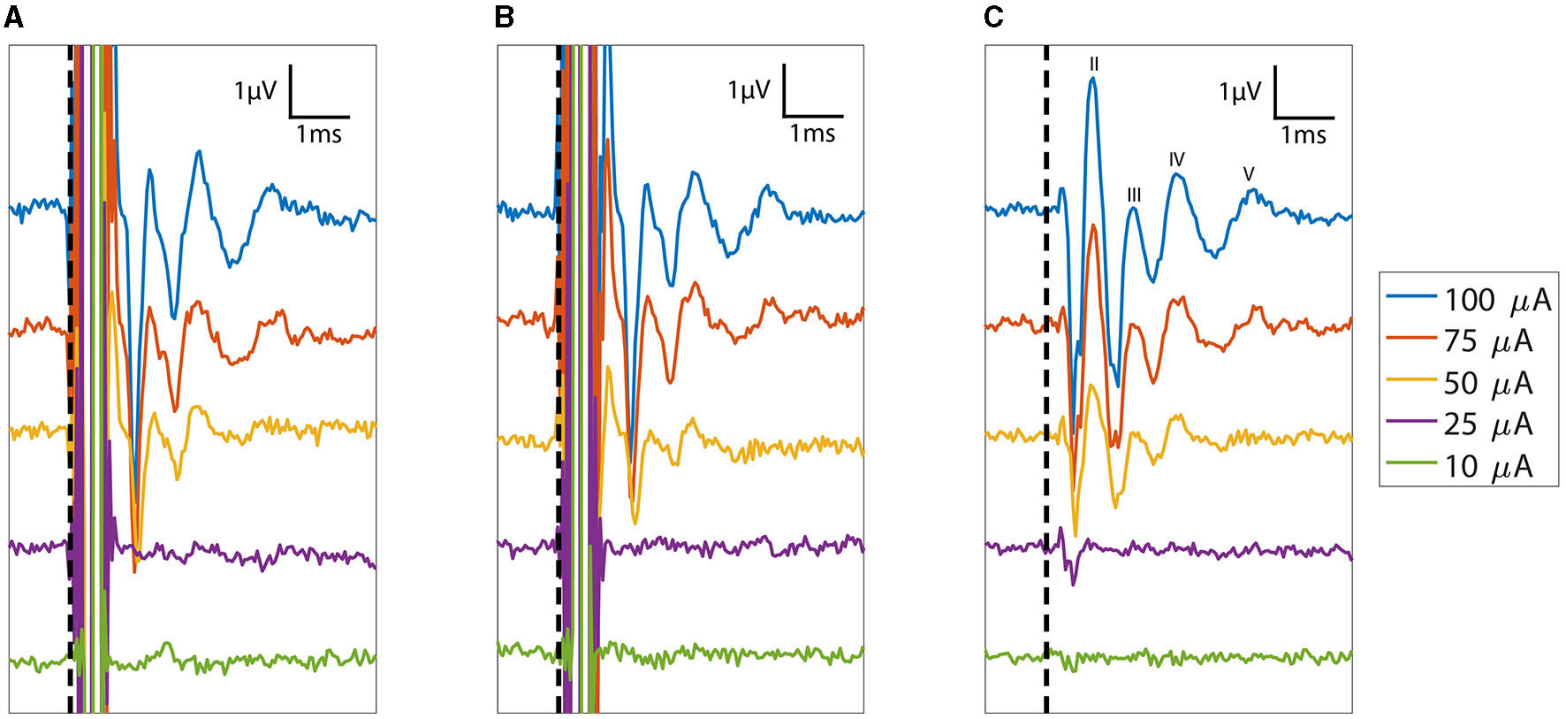
Plot of eABRs (voltage versus post-stimulus latency) collected as a function of stimulation currents from 10 to 100 μA. Response measurements from the cathodic-first and anodic-first begin with the expected large stimulus artifact between 0 and 0.5 ms. The consistent polarity and structure of the eABR peaks across cathodic leading and anodic leading (reversed polarity) stimulation indicate that these responses are not stimulation artifacts. The average of the **(A)** cathodic-first and **(B)** anodic-first responses results in artifact cancellation, allowing the **(C)** common eABR signals to be quantified. eABR peaks II, III, IV, and V are labeled in **(C)**. The vertical dashed line denotes stimulation time (*t* = 0).

eABRs were obtained for at least one electrode site in the last four feline subjects. Across the four cats, we stimulated 14 sites and recorded eABRs on 12 of them, giving a gross yield of 85% (Table 1). The general morphology of the eABR responses was consistent between animals. Representative eABR data collected at stimulation levels ranging from 10 to 100 μA are presented in Figure 6 with offsets of 2 μV between successively higher stimulation levels. The eABR waveform latencies and amplitudes were similar to stereotypical ABRs reported in previous studies using microelectrode arrays for electrical stimulation and in response to acoustic stimulation (Achor and Starr, 1980; Badi et al., 2003; Hillman et al., 2003). The thresholds, which are defined at the first emergence of peak II of the eABR waveform (peak I is likely masked by the electrical artifact corresponding to direct activation of the auditory nerve), varied between implants and stimulation sites but always occurred between 10 and 75 μA, although the range of threshold values was only sparsely explored. These thresholds are consistent with thresholds reported by Badi et al. (2003) and Middlebrooks and Snyder (2007) in prior studies using Utah arrays but higher than those presented with NeuroNexus arrays (10–30 μA). eABRs were observed for both cathodic-first and anodic-first stimulation regimens, with cathodic-first stimulation showing typically higher eABR peak amplitudes.

**Table 1:** A table of eABR yields from four cats following implant of ANI USEA using the transbullar translabyrinthine approach.

**Cat/array**	**Electrodes tested for eABRs**	**Electrodes with eABRs present**	**Yield (%)**
1	5	5	100%
2	2	1	50%
3	2	1	50%
4	5	5	100%

The eABR peak II increased to amplitudes between 1.5 and 4 μV, and the peak latency decreased by 0.1 ms with increasing stimulus levels. Stimulation levels explored in this study did not saturate growth in the peak eABR amplitudes. Stimulation intensities were limited to 100 μA; the maximum demonstrated safe chronic stimulation level for the Utah array used for cortical and peripheral nerve implants (Kim et al., 2015; Rajan et al., 2015; Flesher et al., 2016; Armenta Salas et al., 2018; Caldwell et al., 2020; George et al., 2020). The latency decreases saturated at higher intensities, which is indicative of approaching the maximum recruitment of axons around that electrode site. However, the sampling frequency of our amplifier limited the precise determination of latency changes between minimum and maximum stimulation.

**Figure 7:**
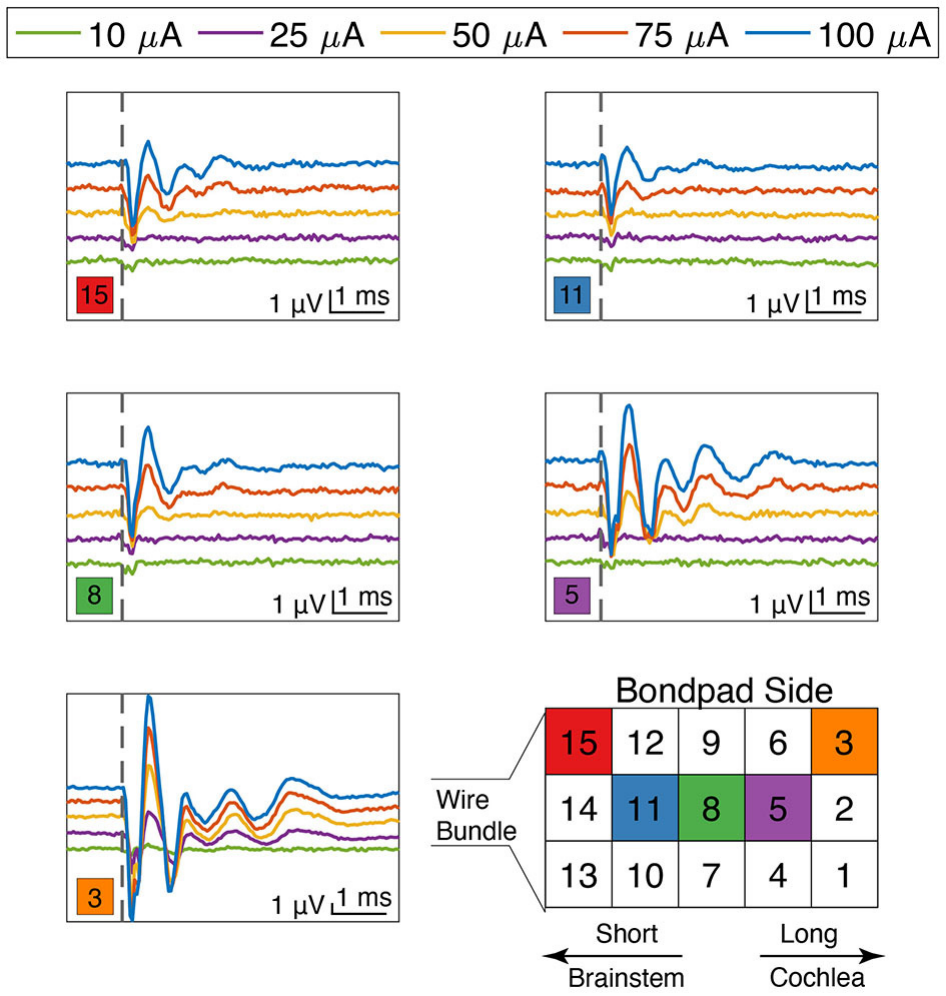
eABR responses for stimulation currents from 10 to 100 μA as a function of electrode site on the array. Electrode positions are denoted by the colored square in the lower left corner near each plot. Thresholds, amplitudes, latencies, and waveform characteristics varied as a function of both stimulation level and electrode site location. The locations of the long and the short shanks are denoted in the array diagram on the bottom right, as well as references to key auditory transduction pathway landmarks. Scale bars are identical for all plots. The vertical dashed line denotes stimulation time (*t* = 0).

The ANI USEA electrode shank lengths increase from 0.5 to 0.9 mm in 0.1 mm increments and are designed to reach different fiber populations across the auditory nerve bundle. Encouragingly, we observed that the waveform shapes and thresholds of the eABR varied with the stimulated electrode sites. Figure 7 shows varying eABR responses for different stimulation sites at different stimulation levels. Across different stimulation sites, there are clear differences in thresholds, peak amplitudes, peak latencies, and fiber recruitments due to the electrode's position on the array. These changes in eABR waveform characteristics between electrode sites resemble those found when examining elicitation of the ABR using acoustic stimuli with varying spectro-temporal content (Bauch et al., 1980). These data could indicate that each electrode is exciting different populations of axons across the tonotopy of the auditory nerve; however, further studies involving direct recordings of neurons across the tonotopic organization of the auditory pathway in response to ANI stimulation are needed to confirm the selectivity of ANI activation. Stimulation with the longest shank was also found to produce eABR signals more commonly than on the shortest shafts. The two sites listed in Table 1 that did not elicit noticeable eABRs with our maximum current level of 100 μA were also short shanks (i.e., site number 14 and 12 respectively), suggesting these sites may not have been inserted sufficiently into the auditory nerve for lower threshold activation. Further, the less robust responses by the shorter electrodes on Figure 7 may indicate that they were not fully implanted.

In terms of potential side effects associated with activating the surrounding nerves, such as vestibular and facial nerves, we did not observe any peaks in the eABR recordings indicative of facial or vestibular nerve activation. We also did not observe any facial twitching or eye movement during stimulation. These observation are encouraging for the ability to elicit auditory activation with the USEA based ANI without current spreading to neighboring nerves, at least for current levels up to 100 μA tested in our experiments.

## 4 Discussion

Direct implantation of a penetrating electrode array into the auditory nerve bundle may provide several important advantages compared to CI electrode arrays. More selective nerve fiber stimulation at lower thresholds and activation of a broader range of frequency fibers may be achieved with ANI stimulation, potentially affording the patients more resolved and broader auditory percepts compared to CI stimulation. The present study evaluated the surgical feasibility of implanting a new type of ANI USEA into the feline auditory nerve to develop a preclinical animal model for future chronic animal work and translate this technology to human patients. The initial positive results of our translabyrinthine approach in achieving favorable impedances and eABR activity motivate further studies for refining and evaluating the long-term stability and function of this USEA technology in chronic cat experiments and in developing new stimulation algorithms appropriate for direct auditory nerve stimulation.

The results from this paper highlight the translabyrinthine approach as a favorable surgical approach for our preclinical feline study leveraging the USEA-based ANI. This surgical approach compromises the semi-circular canals of the vestibular labyrinth to access the internal meatus. The translabyrinthine approach will be justified in the initial patient population that will be implanted with the ANI, which includes those with ossified cochlea or not benefitting from prior cochlear implantation and who already have compromised vestibular function. Development of additional surgical approaches that avoid damaging the vestibular nerves, such as an infralabyrinthine or infracochlear approach, can be considered to address the larger patient population who could benefit from an ANI. Evaluating these surgical approaches for broader patient populations would be difficult in a feline model, as there is not enough space in the feline skull to perform these approaches. However, a non-human primate model with a larger skull could be more beneficial to better evaluate these broad surgical approaches, in addition to further human cadaver experiments.

While the electrophysiological results from this report are encouraging, it is important to highlight that two of the 14 tested stimulation sites did not evoke eABR activity. These two sites had short electrodes and were most likely poorly embedded within the auditory nerve following implantation. Better techniques for inserting and keeping all electrodes embedded in the auditory nerve may need to be investigated to ensure that all electrode sites are functional. Alternatively, making the shorter electrodes longer may allow for the placement of more functional sites securely in the nerve. Additionally, four sites in this study had high-impedance measurements. One of these sites was broken prior to handling. The other three high-impedance sites were short electrodes. These high impedances may stem from bondpad failures, which can occur during surgical manipulation or implant of the device. They are unlikely electrode tip breaks, as the short electrodes do not contact the petrous portion of the temporal bone during implant. The issues with bondpad failure stem were a known issue in the devices manufactured for this study, and the manufacturer has since addressed this failure mode.

## Data availability statement

The original contributions presented in the study are included in the article/supplementary material. Raw data supporting the findings of this study are available upon reasonable request. The sponsor of the study will review each request to verify that no confidential or proprietary data is shared.

## Ethics statement

The animal study was approved by University of Utah Institutional Animal Care and Use Committee and University of Minnesota Institutional Animal Care and Use Committee. The study was conducted in accordance with the local legislation and institutional requirements.

## Author contributions

WT: Formal analysis, Investigation, Writing – original draft, Writing – review & editing, Conceptualization, Methodology. SZ: Investigation, Methodology, Writing – original draft, Writing – review & editing, Conceptualization, Formal analysis. IS: Investigation, Methodology, Writing – original draft, Writing – review & editing, Conceptualization, Formal analysis. ML: Conceptualization, Writing – review & editing. FS: Conceptualization, Writing – review & editing, Supervision. TL: Conceptualization, Writing – review & editing, Supervision. HL: Conceptualization, Methodology, Supervision, Writing – review & editing. DW: Methodology, Supervision, Writing – review & editing, Conceptualization. LR: Conceptualization, Supervision, Writing – review & editing. MA: Methodology, Supervision, Writing – review & editing, Conceptualization.
